# Supplementary material for: Animal Video Lovers Always Have Company: The Role of Cyber-Mediated Animal Attachment in Loneliness
Source: Animals (Basel). 2025 Sep 4;15(17):2593. doi: 10.3390/ani15172593 (PMC12427423; doi:10.3390/ani15172593)
Supplement: Supplementary file 1 [file animals-15-02593-s001.zip › S1 Scale development and revision.pdf]

Supplementary Materials S1: Questionnaire Development

In this study, we developed original scales for online animal video engagement and cyber-mediated animal attachment.

1 Methods

1.1 Procedure

The development of the questionnaire followed a multi-stage procedure.

Step 1: Initial Item Generation

Online animal video engagement was defined as watching, liking, sharing, and commenting on animal-related content across social media and streaming platforms. Based on individual interviews, we identified common behavioral patterns associated with such engagement and generated an initial pool of 7 items.

Cyber-mediated animal attachment refers to a novel form of emotional attachment toward animals shaped by parasocial intimacy via social media. The initial version of the cyber-mediated animal attachment scale was adapted from the Lexington Attachment to Pets Scale (Johnson et al., 1992) and enriched with qualitative insights from interviews, resulting in a preliminary pool of 26 items.

Step 2: Expert Review and Pilot Testing

Experts in psychology and media studies reviewed the initial items for content validity, clarity, and relevance. Revisions were made accordingly to improve item wording and conceptual accuracy. A small-scale pilot test further informed minor refinements.

Step 3: Preliminary Testing and Revision

A preliminary version of the questionnaire was administered to Sample 1 (n = 154). Item analysis and exploratory factor analysis (EFA) were conducted to examine the structure and psychometric properties of the scale, leading to further revision and item reduction.

Step 4: Validation

The revised questionnaire was then administered to Sample 2 (n = 416) for confirmatory factor analysis (CFA) and reliability testing. This step aimed to validate the underlying factor structure and assess the internal consistency of the final scale.

1.2 Participants

Sample 1: Data were collected via an online survey, yielding 154 valid responses. Among the participants, 32 were male (20.78%) and 122 were female (79.22%). A total of 113 participants (73.4%) were single, while 41 (26.6%) were in a relationship. Regarding pet ownership, 42 participants (27.27%) were currently or previously pet owners, and 112 (72.73%) had never owned a pet. This sample was used for preliminary item analysis and exploratory factor analysis (EFA).

Sample 2: Data were collected through both online and offline surveys, resulting in 416 valid responses. The sample included 150 males (36.1%) and 266 females (63.9%). Of these, 288 (69.2%) were single and 128 (30.8%) were non-single. A total of 142 participants (34.1%) were currently or previously pet owners, and 274 (65.9%) had never owned a pet. The average age was 23.2 years (SD = 1.953). This sample was used for reliability testing and confirmatory factor analysis (CFA).

2 Results

Data were analyzed using SPSS 26.0 for item analysis, discrimination testing, exploratory factor analysis (EFA), and internal consistency reliability (Cronbach's alpha). Confirmatory factor analysis (CFA) was conducted using Mplus to assess the structural validity of the scales.

2.1 Online Animal Video Engagement Questionnaire

2.1.1 Item Analysis

Based on the total scale scores from Sample 1 (n = 154), participants were divided into high-score and low-score groups using the top 27% and bottom 27% as cutoff points, following conventional statistical practice. The critical value for the low-score group was 2.25, and for the high-score group, it was 3.71. An independent samples t-test was conducted to compare the responses of the high and low groups for each item (see Table 1). Results indicated that all item mean differences between the groups were statistically significant ($p < 0.001$), suggesting good discrimination across items.

Table S1 Independent Samples t-Test for High and Low Groups on Online Animal Video Engagement Items (n = 154)

Item	*t*	*df*	*p*	Item	*t*	*df*	*p*
Q1	16.71	52.6	<0.001	Q5	20.49	75	<0.001
Q2	9.25	52.3	<0.001	Q6	21.06	75	<0.001
Q3	17.7	75	<0.001	Q7	6.10	53.03	<0.001
Q4	16.66	75	<0.001				

Next, item-total correlations were calculated. Items with correlation coefficients below 0.40 were considered for deletion. The overall Cronbach's α coefficient for the scale was 0.863. Item 7 had an item-total correlation below 0.40, and its removal increased the α coefficient above 0.863; therefore, it was deleted. All the remaining items met the retention criteria (see Table 2).

Table S2 Item-Total Correlations for the Online Animal Video Engagement Scale (n = 154)

Item	Item-Total Correlation	Cronbach's α if Item Deleted
Q1	0.685	0.837
Q2	0.595	0.850
Q3	0.665	0.840
Q4	0.660	0.841
Q5	0.742	0.828
Q6	0.761	0.825
Q7	0.338	0.879

2.1.2 Exploratory Factor Analysis

To determine whether the data from Sample 1 were suitable for exploratory factor analysis (EFA), the Kaiser–Meyer–Olkin (KMO) measure and Bartlett's test of sphericity were conducted. The results showed a KMO value of 0.868, and Bartlett's test was significant, indicating that the sample data were appropriate for EFA (see Table 3).

Table S3 Results of Bartlett's Test of Sphericity for Online Animal Video Engagement Scale (n = 154)

KMO Measure of Sampling Adequacy		0.868
Bartlett's Test of Sphericity	Approx. Chi-Square	444.047
	df	15
	Sig.	<0.001

Exploratory factor analysis (EFA) was conducted using principal component analysis with varimax rotation. The item deletion criteria for this study included (1) communality < 0.40; (2) factor loading < 0.40; (3) high and similar loadings on multiple factors; and (4) fewer than three items in a factor. Based on the theoretical framework referenced during item development and the conceptual design of the questionnaire, only one factor was ultimately extracted, which explained 63.68% of the total variance. The final version of the Online Animal Video Engagement Scale comprised six items (see Table 4).

Table S4 Factor Loadings and Communalities of the Online Animal Video Engagement Scale (n = 154)

Item	Factor Loading	Communality
	F1	
**Q5** How often do you talk to others about your favorite online animals?	0.834	0.696
**Q6** Do you actively search for information about your favorite online animals?	0.832	0.692
**Q1** How frequently do you watch animal videos?	0.806	0.650
**Q3** How frequently do you like animal videos?	0.803	0.646
**Q4** How frequently do you share your favorite online animal videos with others?	0.795	0.633
**Q2** How frequently do you comment under animal videos?	0.658	0.433

2.1.3 Reliability Analysis

A reliability analysis was conducted using Sample 2 (n = 416). The Online Animal Video Engagement Scale demonstrated good internal consistency, with a Cronbach's α coefficient of 0.819.

2.1.4 Confirmatory Factor Analysis

Following the exploratory factor analysis, confirmatory factor analysis (CFA) was performed on Sample 2 using Mplus, testing a single-factor model. The model fit indices are presented in Table 5. A Comparative Fit Index (*CFI*) and Tucker–Lewis Index (*TLI*) greater than 0.90 indicate good fit; a Root Mean Square Error of Approximation (*RMSEA*) less than 0.08 indicates reasonable fit, and values under 0.10 are considered acceptable. A Standardized Root Mean Square Residual (*SRMR*) below 0.05 suggests a good fit, and a *chi-square/df* (*CMIN/df*) ratio between 1 and 3 indicates a good fit, with values under 5 still acceptable. The results indicated an acceptable model fit. The standardized factor structure is shown in Figure 1.

Table S5 Model Fit Indices for the Cyber-mediated animal Attachment Scale (n = 416)

CMIN	*df*	*CFI*	*TLI*	*RMSEA*	*SRMR*	*CMIN/df*
39.95	9	0.959	0.932	0.091	0.03	4.43

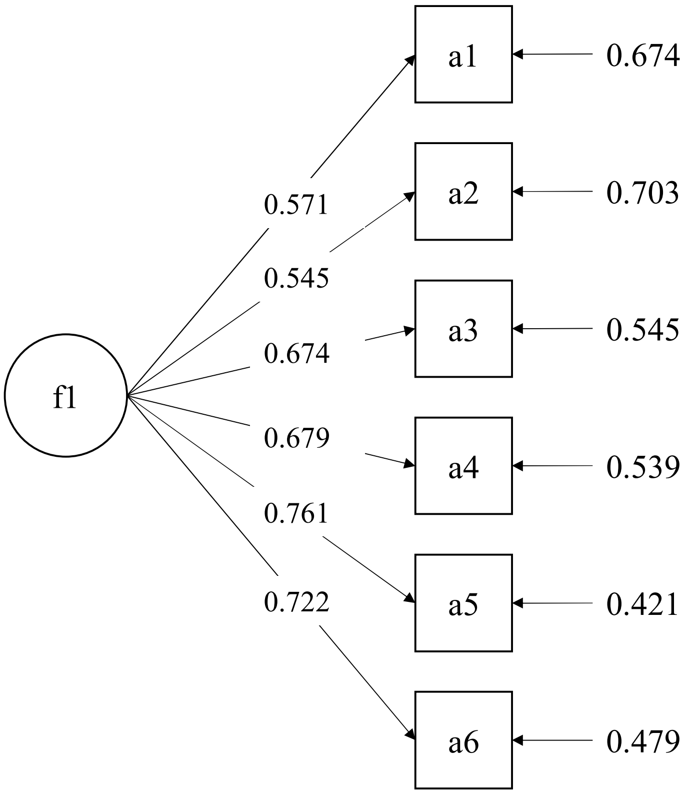

Figure S1 Confirmatory Factor Analysis Model of Online Animal Video Engagement

2.2 Cyber-mediated animal Attachment Questionnaire

2.2.1 Item Analysis

Based on the total scores of Sample 1, participants were divided into high-score and low-score groups using the upper 27% and lower 27% percentile method. The cutoff score for the low group was 2.57, and for the high group, it was 3.19. An independent samples t-test was conducted between the two groups to examine whether each item could effectively distinguish between high and low scorers (see Table 6). The results indicated that Item 20 had an absolute t-value less than 3 and a p-value greater than 0.05, suggesting no significant difference; therefore, it was removed.

Table S6 Independent Samples t-Test for Cyber-mediated Animal Attachment Items (n = 154)

Item	t	df	p	Item	t	df	p
Q1	6.35	72	<0.001	Q14	8.05	39.25	<0.001
Q2	4.61	72	<0.001	Q15	8.54	49.64	<0.001
Q3	5.53	56.04	<0.001	Q16	5.99	72	<0.001
Q4	5.88	48.51	<0.001	Q17	5.00	72	<0.001
Q5	6.09	59.06	<0.001	Q18	10.19	72	<0.001
Q6	3.72	47.25	0.001	Q19	8.8	81	<0.001
Q7	3.56	71	0.001	Q20	0.72	72	0.47
Q8	7.97	72	<0.001	Q21	5.35	67.89	<0.001
Q9	5.14	45.67	<0.001	Q22	5.56	46.06	<0.001
Q10	8.98	72	<0.001	Q23	11.73	72	<0.001
Q11	7.53	43.13	<0.001	Q24	7.39	72	<0.001
Q12	7.86	54.12	<0.001	Q25	10.87	72	<0.001
Q13	11.73	72	<0.001	Q26	6.64	72	<0.001

Item-total correlations were calculated to evaluate the discrimination power of each item. Items with item-total correlation coefficients below 0.40 were removed. As a result, Items 2, 6, 7, 9, and 17 were excluded from the scale. A total of 21 valid items were retained (see Table 7).

Table S7 Item-Total Correlation for Cyber-mediated Animal Attachment (n = 154)

Item	Item-Total Correlation	Cronbach's α if Item Deleted	Item	Item-Total Correlation	Cronbach's α if Item Deleted
Q1	0.459	0.902	Q14	0.598	0.899
Q2	0.368	0.904	Q15	0.543	0.900
Q3	0.452	0.902	Q16	0.403	0.903
Q4	0.452	0.902	Q17	0.399	0.903
Q5	0.468	0.902	Q18	0.634	0.898
Q6	0.303	0.904	Q19	0.570	0.899
Q7	0.322	0.905	Q21	0.420	0.903
Q8	0.586	0.899	Q22	0.502	0.901
Q9	0.353	0.904	Q23	0.648	0.897
Q10	0.582	0.899	Q24	0.555	0.900
Q11	0.475	0.902	Q25	0.661	0.897
Q12	0.482	0.901	Q26	0.493	0.901
Q13	0.657	0.897			

2.2.2 Exploratory Factor Analysis

First, the suitability of Sample 1 for factor analysis was examined according to standard statistical criteria (see Table 8). The results showed that the Kaiser–Meyer–Olkin (KMO) measure was 0.883, and Bartlett's test of sphericity was

significant ($p < 0.01$), indicating that the data were appropriate for conducting an exploratory factor analysis (EFA) to examine the underlying structure of the questionnaire.

Table S8 Results of Bartlett's Test of Sphericity for Cyber-mediated Animal Attachment Scale (*n* = 154)

**KMO Measure of Sampling Adequacy**		**0.883**
Bartlett's Test of Sphericity	Approx. Chi-Square	1462.55
	df	190
	Sig.	<0.001

Exploratory factor analysis was performed using principal component analysis and maximum orthogonal rotation. The following criteria were used for item deletion: (1) communalities < 0.4; (2) factor loadings < 0.4; (3) items with high loadings on multiple factors and close factor values; (4) factors with fewer than three items. Items that did not meet these criteria were gradually deleted. Based on the theoretical framework referenced during the development of the items and the design of the questionnaire for this study, three factors were ultimately extracted, explaining 64.348% of the total variance. As a result, the cyber-mediated animal attachment scale was refined into three dimensions and 12 items (see Table 9).

Table S9 Factor Loadings and Communalities of the Cyber-mediated Animal Attachment Scale (*n*=154)

**Dimension**	**Item**	**Factor Loading**			**Communality**
		**F1**	**F2**	**F3**	
Emotional Attachment	**Q16** I feel happy when watching pet videos.	0.847			0.733
	**Q11** If the online pet I like were my pet, I would do my best to take care of it.	0.803			0.696
	**Q12** I find the online pet I like very attractive.	0.761			0.619
	**Q4** Watching pet videos increases my sense of happiness.	0.737			0.597
	**Q14** I feel hurt when the online pet I like gets sick or passes away.	0.710			0.637
Significance Attachment	**Q19** The way others treat the online pet I share affects my impression of them.		0.815		0.706
	**Q22** I feel hurt when my friends mock the online pet I like.		0.784		0.660
	**Q23** I feel uncomfortable when I can't watch videos of my favorite online pet for a few days.		0.751		0.743
	**Q18** Pet videos are essential in my life.		0.714		0.638
Intimacy Attachment	**Q10** I feel close to the online pet I like.			0.768	0.645
	**Q5** I think the online pet I like is special.			0.697	0.543
	**Q3** I can understand the emotions of the online pet I like.			0.672	0.505

Emotional Attachment refers to the subjective emotional experience an individual has when watching online pet videos, where the individual is emotionally moved by the pet. Significance Attachment reflects how important the individual perceives the online pet to be. Since the formation of cyber-mediated animal attachment is based on individual fantasies, the third dimension is Intimacy Attachment, which is defined as the perceived closeness to the online pet. This means the individual is able to sense the pet's emotions, creating a sense of intimacy.

2.2.3 Reliability Analysis

Reliability analysis was conducted on Sample 2 (see Table 10), calculating Cronbach's α coefficients for the three dimensions as well as the overall scale. The coefficients for the overall scale, the Significance Attachment dimension, and the Emotional Attachment dimension were all greater than 0.7, indicating high internal consistency. The coefficient for the Intimacy Attachment dimension ranged between 0.6 and 0.7. As noted by Hair Jr et al. (2010), when the number of items in a dimension is fewer than six, a Cronbach's α coefficient greater than 0.6 suggests that the scale is reliable. Given that the Intimacy Attachment dimension only contains three items, the reliability is considered acceptable.

Table S10 Internal Consistency Coefficients for the Cyber-mediated Animal Attachment Scale (n=416)

**Dimensions**	**Significance**	**Emotional**	**Intimacy**	**Total**
Cronbach's α	0.739	0.795	0.602	0.835

2.2.4 Confirmatory Factor Analysis

After completing the exploratory factor analysis, Sample 2 was used for confirmatory factor analysis, and Mplus was employed for model fitting based on the three dimensions: Emotional Attachment, Intimacy Attachment, and Significance Attachment (see Table 11). The results showed that *CFI* and *TLI* values greater than 0.9 indicate a good model fit. *RMSEA* values less than 0.08 suggest a reasonable model fit, with values less than 0.1 being acceptable. *SRMR* values less than 0.05 indicate a good fit, with values under 0.08 still acceptable. *CNIN/df* values between 1 and 3 are considered good, and values below 5 indicate an acceptable model fit. Based on the results, the model fit was deemed acceptable, and the structural diagram is shown in Figure 2.

Table S11 Model Fit Indices for the Cyber-mediated Animal Attachment Model (n=416)

CMIN	*df*	*CFI*	*TLI*	*RMSEA*	*SRMR*	*CMIN/df*
141.48	51	0.935	0.915	0.065	0.05	2.77

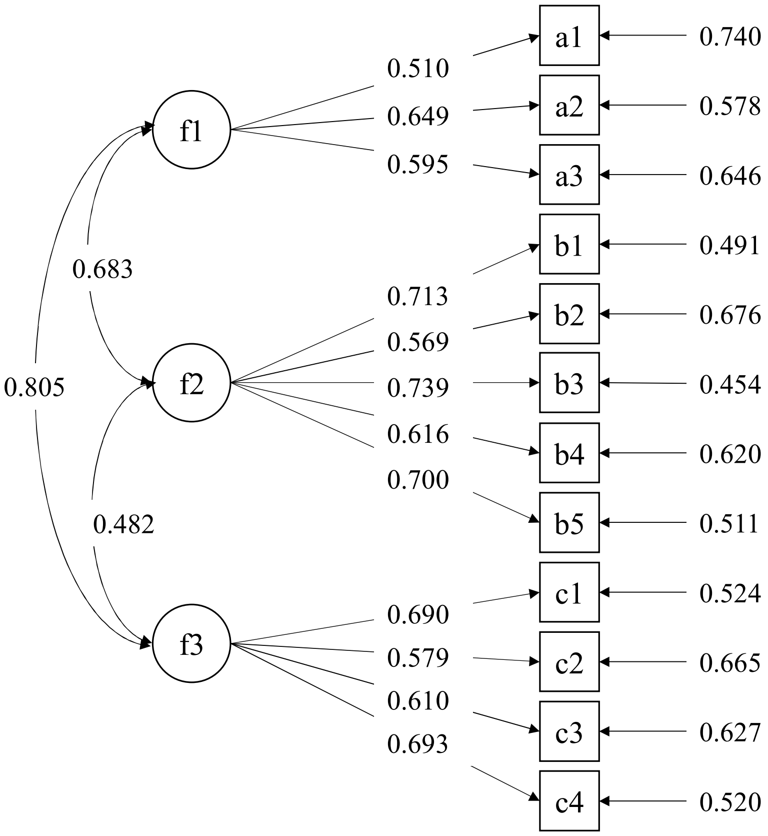

Figure S2 Confirmatory Factor Analysis Model of Cyber-mediated Animal Attachment Scale

References

Hair Jr, J. F., Black, W. C., Babin, B. J., & Anderson, R. E. (2010). Multivariate data analysis. In *Multivariate data analysis* (pp. 785-785).

Johnson, T. P., F., G. T., & and Stallones, L. (1992, 1992/09/01). Psychometric Evaluation of the Lexington Attachment to Pets Scale (Laps). *Anthrozoös,* *5*(3), 160-175. https://doi.org/10.2752/089279392787011395
